# Methionine Oxidation Changes the Mechanism of Aβ Peptide Binding to the DMPC Bilayer

**DOI:** 10.1038/s41598-019-42304-9

**Published:** 2019-04-11

**Authors:** Christopher Lockhart, Amy K. Smith, Dmitri K. Klimov

**Affiliations:** School of Systems Biology, George Mason University, Manassas, VA 20110 USA

## Abstract

Using all-atom explicit solvent replica exchange molecular dynamics simulations with solute tempering, we study the effect of methionine oxidation on A*β*10–40 peptide binding to the zwitterionic DMPC bilayer. By comparing oxidized and reduced peptides, we identified changes in the binding mechanism caused by this modification. First, Met35 oxidation unravels C-terminal helix in the bound peptides. Second, oxidation destabilizes intrapeptide interactions and expands bound peptides. We explain these outcomes by the loss of amphiphilic character of the C-terminal helix due to oxidation. Third, oxidation “polarizes” A*β* binding to the DMPC bilayer by strengthening the interactions of the C-terminus with lipids while largely releasing the rest of the peptide from bilayer. Fourth, in contrast to the wild-type peptide, oxidized A*β* induces significantly smaller bilayer thinning and drop in lipid density within the binding footprint. These observations are the consequence of mixing oxidized peptide amino acids with lipids promoted by enhanced A*β* conformational fluctuations. Fifth, methionine oxidation reduces the affinity of A*β* binding to the DMPC bilayer by disrupting favorable intrapeptide interactions upon binding, which offset the gains from better hydration. Reduced binding affinity of the oxidized A*β* may represent the molecular basis for its reduced cytotoxicity.

## Introduction

Progression of Alzheimer’s disease is related to the accumulation of cytotoxic A*β* peptides^1^, which are naturally cleaved from membrane-spanning *β*-amyloid precursor proteins by *β* and *γ* secretases^2^. This process results in the extracellular release of several A*β* alloforms, of which the 40-residue monomer A*β*1–40 is most prevalent^3^. Over time, A*β* peptides aggregate to form higher-ordered oligomers capable of inducing neuronal death^2^. The cytotoxicity of A*β* is purportedly related to its interactions with cellular membranes, which may increase calcium permeation eventually initiating neuron apoptosis^4^. Depending on the concentration, A*β* can reside on membrane surface in monomeric^5^ or oligomeric forms^6^.

The A*β* amino acid sequence contains a single methionine residue at position 35. Being sensitive to reactive oxygen species, methionine can readily oxidize into methionine sulfoxide (MetO) by incorporating an oxygen atom into its side chain^7,8^. Because A*β* is normally localized to brain tissues, which consume 20% of the body’s oxygen, it has a high propensity to become oxidized, most likely mediated by Cu^2+^ ions^9^. Consequently, it has been estimated that oxidized A*β* species represent from 10 to 50% of all A*β* peptides found in the brain^10,11^. Although oxidized methionine side chains may adopt two diastereomeric forms, R and S, with slightly different hydrogen bonding properties, there is no evidence for natural preference of either of the two^7,12^. Most experimental studies indicate that, compared to wild-type (WT) A*β*, methionine oxidation impairs formation of aggregated A*β* species^13–15^. This outcome is predominately associated with a decreased rate of assembly^14,16^ delaying cytotoxic effects^17,18^. However given enough time to aggregate, oxidized A*β* peptides have been reported to approximately match the cytotoxicity of WT A*β*^9,19^.

Although previous experimental and computational studies have mostly focused on WT A*β*^20,21^, few have also explored structural consequences of methionine oxidation for A*β* monomers in aqueous environment. Specifically, solution NMR spectroscopy has been used to investigate the effect of methionine oxidation on A*β* peptides in water^22^. In general, oxidation produced a more flexible peptide C-terminus by increasing its backbone mobility and preventing the G37–G38 turn. Qualitatively similar conclusions were drawn from replica exchange molecular dynamics simulations^23^, where WT peptides were found to be more structured than those oxidized. Constant temperature molecular dynamics simulations at physiological conditions have further suggested that oxidation increases the solvent accessible area of the C-terminus^24^. CD and NMR spectroscopy have been used to determine the structure of WT and methionine-oxidized A*β* in a membrane-mimicking water-SDS environment^12,14^. These studies have revealed a reduced helical propensity in oxidized A*β*1–40 compared to its WT counterpart. Collectively, most of previous investigations argue that the overall effect of methionine oxidation, regardless of local environment, is a disordering in the peptide C-terminus and increase in its structural fluctuations. It is worth noting that methionine oxidation also determines the polymorphic form of A*β*1–42 fibrils^25–27^.

To date, a detailed study elucidating the molecular mechanisms of binding of the methionine-oxidized A*β* peptide to cellular membranes has not been performed. Nonetheless, given the natural prevalence of oxidized A*β* species, the corresponding investigation would be highly relevant to Alzheimer’s molecular pathogenesis. To bridge this gap, we performed isobaric-isothermal all-atom replica exchange molecular dynamics simulations with solute tempering (REST)^28^ to probe the binding of oxidized A*β* peptides to the DMPC bilayer. As a control, we utilized our previous studies, which probed binding of WT A*β* peptides to the same bilayer^29,30^. Below we present our analysis, which delineates the MetO binding mechanism and distinguishes it from that governing WT binding. We have also determined the affinities of MetO and WT peptides binding to the DMPC bilayer and compared our simulation results against experimental findings.

## Methods

### All-atom model and simulation details

**Figure 1:**
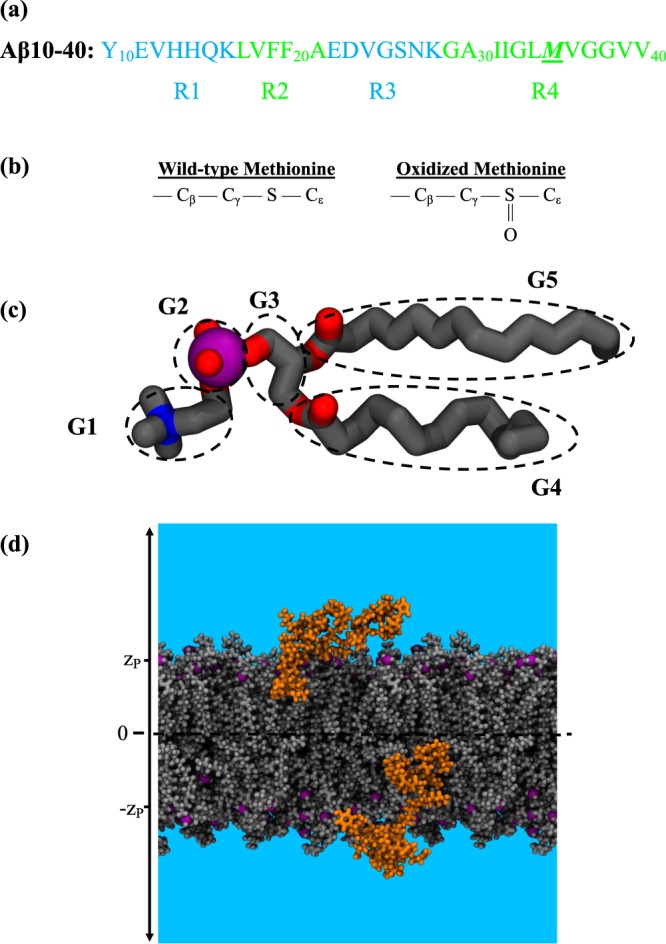
(**a**) Sequence of A*β*10–40 peptide with highlighted regions R1–R4. (**b**) Oxidation of hydrophobic methionine side chain results in polar methionine sulfoxide. (**c**) A DMPC lipid is divided into five structural groups: choline (G1), phosphate (G2), glycerol (G3), and two fatty acid tails (G4 and G5). The lipid headgroup is composed of three polar groups G1–G3. The bilayer hydrophobic core is formed by the tails G4 and G5. Phosphorus, oxygen, and nitrogen atoms are in purple, red, and blue, respectively. (**d**) A simulation snapshot illustrating binding of oxidized MetO A*β* peptides to the opposite leaflets of DMPC bilayer. The centers of mass of lipid phosphorus P atoms in each leaflet are approximately restrained at ±*z*_*P*_. Water is shown in blue. For clarity, we omitted ions.

We have studied the binding of methionine oxidized (MetO) A*β*10–40 monomers to the DMPC bilayer (Fig. 1). To understand the impact of methionine oxidation on the binding mechanism, we compared our results against previous simulations of wild-type (WT) A*β*10–40 monomers binding to the same bilayer^29^. The amino-truncated A*β*10–40 monomer was chosen as a proxy to full-length A*β*1–40. Our previous simulations^29,30^ have indicated that this peptide reproduces several A*β*1–40 experimental observations, including the distribution of helical structure along the sequence and partial insertion of A*β* in the bilayer^12,31,32^. Additionally, A*β*10–40 peptides are naturally occurring cytotoxic and amyloidogenic species^33–36^. The DMPC bilayer was chosen because it is well characterized experimentally affording direct comparisons with simulation data^30^ and the binding of WT A*β*10–40 to DMPC bilayer approximately reproduces experimental observations for SDS micelles^12,31^.

Our simulation system consisted of 98 DMPC lipids forming a bilayer solvated by 4356 water molecules. Two sodium ions were introduced to compensate A*β* charge. A*β* N- and C-termini were acetylated and amidated, respectively. A*β* peptides were placed on either side of the lipid bilayer to take advantage of its symmetry and ensure that both leaflets approximately featured the same mass and pressure profiles^37^ (Fig. 1d). The initial size of the simulation box was 65 Å × 65 Å × 77 Å. Peptides were modeled using the CHARMM22 force field with CMAP corrections^38^, whereas lipids were modeled with the CHARMM36 force field. Water was represented with the modified TIP3P model^39,40^. The force field parameters for oxidized methionine were derived from DMSO parameters^41^ following previously published reports^42^. The force field topology and diastereomeric form were adopted from the study of Jas and Kuczera^43^. The resulting methionine sulfoxide was the R diastereomer, which apparently exhibits higher propensity to engage in hydrogen bonding^12^.

Molecular dynamics simulations were performed using NAMD^44^, which employed a 1 fs integration time step and periodic boundary conditions. Electrostatic interactions were computed using particle-mesh Ewald summation, and van der Waals interactions were smoothly switched off in the interval from 8 to 12 Å using the force switch option. Temperature was held constant using Langevin dynamics with a damping coefficient of 5 ps^−1^. Pressure was controlled via a Langevin piston method applying a semi-isotropic scheme, which results in independent fluctuations in the bilayer plane and normal. Because our simulation system included the DMPC bilayer, which could potentially disintegrate at high REST temperatures (see below), we implemented two sets of restraints to ensure bilayer integrity. The first harmonically restrained the center of mass of lipid phosphorus (P) atoms in a leaflet to the position *z*_*P*_ = 17.35 Å with a force constant *k* = 6.5 kcal/mol/Å^2^. This force constant value was derived from restraint-free control simulations of the DMPC bilayer without A*β* peptides requiring that the restraints do not distort fluctuations of P atoms^29^. The second set of restraints affects A*β* or ion atoms when they come within 4 Å of the *z* periodic boundary (the axis normal to the bilayer plane). These were implemented as harmonic restraints with force constant *k* = 10 kcal/mol/Å^2^ and sought to inhibit interpeptide interactions. The contribution of all restraints to the potential energy at 330 K is minor (≈0.6 kcal/mol $\lesssim$ *RT*). Moreover, our previous studies have shown that the effect of restraints on lipid structural properties is generally within the sampling error^30,45^. Because we detected no instances of interpeptide interactions via amino acid side chain contacts or cross-bridges by a third molecule, we assume that the peptides bind to the bilayer independently as monomers.

Conformational sampling was performed using isobaric-isothermal replica exchange molecular dynamics simulations with solute tempering (REST)^28^. Because REST formalism is described in detail elsewhere^28,46^, here we present its brief overview. We considered $R=8$ replicas distributed geometrically from temperature ${T}_{0}=330\,{\rm{K}}$ to ${T}_{R-1}=430\,{\rm{K}}$. An exchange between replicas *r* and $r+1$ occurs with the probability $\omega =\,{\min }\,[1,{e}^{-{\rm{\Delta }}}]$, where ${\rm{\Delta }}={\beta }_{r}({H}_{r}({X}_{r+1})-{H}_{r}({X}_{r}))$ + ${\beta }_{r+1}({H}_{r+1}({X}_{r})-{H}_{r+1}({X}_{r+1}))$, $\beta ={(\bar{R}T)}^{-1/2}$, *H* is the enthalpy, and *X* defines a system configuration. The solvent-solvent and solute-solvent interactions in replica *r* with the temperature *T*_*r*_ were scaled by the factors ${T}_{r}$/${T}_{0}$ and ${({T}_{r}/{T}_{0})}^{1/2}$, respectively. Because these scaling factors eliminate from $\omega$ the solvent-solvent energy contribution, the number of replicas can be reduced without affecting the temperature range or exchange rates. Following our previous studies^46,47^ A*β* peptides and two ions were treated as solute, whereas lipids and water were considered as solvent. In our simulations, exchanges were attempted every 2 ps with the probability of success of 0.21. In total, we generated five REST trajectories collecting in all 0.8 μs of sampling (100 ns per replica). Initial 3.8 ns of sampling per replica in each trajectory were discarded as unequilibrated reducing the total equilibrated sampling to 648 ns. However, due to independent binding of two A*β* peptides to the DMPC bilayer, the effective sampling per peptide is doubled to almost 1.3 μs. Importantly, our previous work has shown that REST reproduces well the conformational sampling of A*β*10–40 binding to the bilayer collected via full-scale replica exchange molecular dynamics^46^.

In addition, we performed separate REST simulations probing the conformational ensemble of the MetO A*β* monomer in lipid-free water. In short, this system contained 4959 water molecules and a single sodium ion. The REST simulations utilized $R=8$ replicas distributed geometrically within the temperature interval from ${T}_{0}=330\,{\rm{K}}$ to ${T}_{R-1}=440\,{\rm{K}}$. In all, five REST trajectories were produced collecting 0.8 μs of sampling (100 ns per replica). Initial 2 ns of REST sampling per replica were excluded as unequilibrated reducing the total equilibrated sampling to 720 ns.

### Computation of structural probes

Molecular interactions were quantified by the contacts occurring between A*β* amino acid side chains and lipid structural groups G1–G5 (Fig. 1c). To detect contacts, the positions of side chains or groups were represented by the respective centers of mass. Two amino acids or an amino acid and a lipid group were in contact if the distance between them was less than 6.5 Å. To facilitate analysis, the A*β*10–40 sequence was broken up into the N-terminal region R1 (residues 10–16), central hydrophobic cluster R2 (residues 17–21), turn region R3 (residues 22–28), and hydrophobic C-terminal R4 (residues 29–40) (Fig. 1a). A*β* secondary structure was computed using STRIDE^48^. An amino acid was considered in a helical state if it sampled *α*-, 3_10_-, or *π*-helix. A hydrogen bond (HB) between donor (D) and acceptor (A) was formed if the distance ${r}_{DA}\le 3.5\,\AA$ and the angle ∠$DHA\ge {120}^{\circ }$.

To probe the localization of A*β* peptide and lipid structure, we defined several functions. The first is the probability *P*(*z*; *i*) for an amino acid *i* to be found at a distance *z* from the bilayer midplane. Using *P*(*z*; *i*) we computed the equilibrium positions of amino acids *i* along the *z* axis, $\langle z(i)\rangle$. The insertion probability *P*_*i*_(*i*) was derived from *P*(*z*; *i*) as the probability for amino acid *i* to reside below the average position of the center of mass of phosphorus atoms *z*_*P*_. The surface binding probability *P*_*s*_(*i*) is defined as the probability for *i* to be localized in the lipid headgroup region (${z}_{p} < z < {z}_{P}+6.5\,\AA$). The unbinding probability *P*_*u*_(*i*) represents unbound states with $z > {z}_{P}+6.5\,\AA$. The second function is the two-dimensional number density of the bilayer lipid heavy atoms *n*_*l*_(*r*, *z*) computed as a function of the distance *r* to the A*β* center of mass and the distance *z* to the bilayer midplane. In these calculations, *r* and *z* were binned with the interval ${\rm{\Delta }}r={\rm{\Delta }}z=1\,\AA$. One-dimensional number densities for lipid, peptide, and water atoms were obtained in a similar way. Ordering in lipid fatty acid tails was quantified by the lipid carbon-deuterium order parameter *S*_*CD*_ and tail tilts *γ* computed for *sn* − 2 chains^30^.

Thermodynamic quantities are denoted by brackets 〈…〉. Unless otherwise noted, all quantities were computed at 330 K using the weighted histogram analysis method^49^ derived for REST simulations^46^. The entropy *S* of a solute (DMPC bilayer with bound A*β* peptides or A*β* peptide in water) was estimated using Gibbs formula $S=-\bar{R}\,{\sum }_{E}\,P(E){\rm{l}}{\rm{n}}\,P(E)$, where *P*(*E*) is the probability of observing the solute energy *E* computed from weighted histogram analysis. The bin size for *E* was set to 1 kcal/mol. As expected the difference in *S* between MetO and WT systems is approximately independent of bin size. Simulation convergence is analyzed in the Supplementary Information. Reported uncertainties are standard errors about the mean calculated from *n* = 5 trajectories.

## Results

### Effect of oxidation on A*β* structure

**Figure 2:**
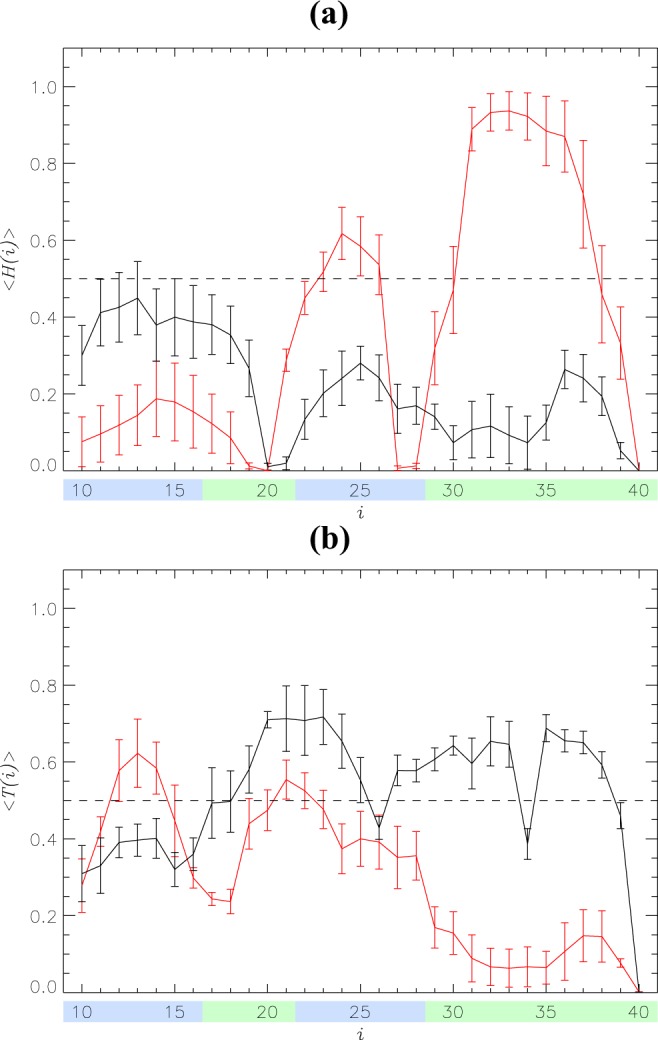
Secondary structure distribution in A*β* peptides. Helical $\langle H(i)\rangle$ and turn $\langle T(i)\rangle$ propensities are shown for amino acids *i* in panels (a and b). The data in black and red correspond to oxidized and reduced A*β* peptides bound to the DMPC bilayer. Vertical bars represent the standard error about the mean calculated from *n* = 5 trajectories. Regions R1–R4 are colored according to Fig. 1a. Methionine oxidation disrupts helical structure but elevates turn propensity.

Our REST simulations explored the equilibrium binding of methionine oxidized (MetO) A*β*10–40 peptides to the DMPC bilayer. To gain insight into the changes in binding mechanism attributed to oxidation, we compare binding of MetO A*β*10–40 to the binding of wild-type (WT) A*β*10–40 to the same bilayer studied by us previously^29^. We first consider the effect of oxidation on A*β* secondary structure. The probabilities for MetO A*β* to adopt helix, turn, and random coil structure are $\langle H\rangle =0.22\pm 0.02$, $\langle T\rangle =0.53\pm 0.02$, and $\langle RC\rangle =0.25\pm 0.02$, respectively. For comparison, in WT A*β*, the corresponding probabilities were 0.39 ± 0.03, 0.30 ± 0.03, and 0.31 ± 0.01. Therefore, methionine oxidation reduces helix content approximately two-fold, almost doubles turn fraction, and moderately reduces random coil structure (a 1.2-fold difference). These structural changes are further detailed in Fig. 2, which presents the distributions of secondary structure along the sequence. To aid the analysis, a secondary structure is assumed formed at amino acid *i* if its probability of occurrence exceeds 0.5. In MetO A*β*, there are no helical amino acids, whereas 18 residues (19–25, 27–33, 35–38) feature turn. For comparison, the WT peptide contains 11 helical residues (23–26 and 31–37), but only 5 turn residues (12–14, 21, and 22). Additionally, two residues (34, 40) in MetO A*β* adopt random coil conformations in contrast to 11 in WT A*β* (10, 16–20, 27–29, 39, and 40). These observations are supported by Table S1, which displays the fractions of helix, turn, and random coil within A*β* regions R1–R4. Indeed, in the WT a stable (namely, helical) structure is observed only in the R4 C-terminus, whereas three regions R2–R4 of MetO A*β* are dominated by turn conformations. Thus, we observe a striking differences in the WT and MetO secondary structure propensities. Collectively, our results imply that Met35 oxidation disrupts helical structure, particularly in the C-terminus, replacing it with turn conformations.

**Figure 3:**
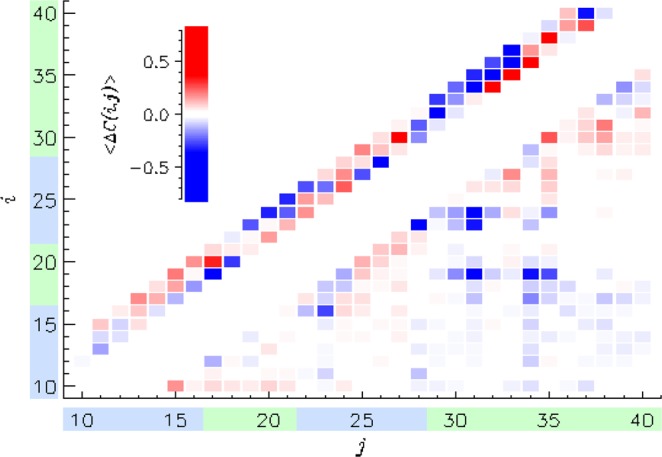
The difference contact map $\langle {\rm{\Delta }}C(i,j)\rangle$ visualizes changes in intrapeptide interactions induced by methionine oxidation. We define $\langle {\rm{\Delta }}C(i,j)\rangle =\langle C(i,j)\rangle -{\langle C(i,j)\rangle }_{WT}$, where $\langle C(i,j)\rangle$ and ${\langle C(i,j)\rangle }_{WT}$ are the contact maps obtained for MetO and WT^29^ A*β*, respectively. The values of $\langle {\rm{\Delta }}C(i,j)\rangle$ are color-coded in the inset scale. Short-range ($|i-j| < 5$) and long-range ($|i-j|\ge 5$) contacts are placed above and below the diagonal. Regions R1–R4 are colored following Fig. 1a. Methionine oxidation generally weakens intrapeptide interactions, while strengthening some local contacts.

We next analyze A*β* tertiary fold. The average number of amino acid contacts $\langle C\rangle$ is 22.7 ± 0.6 in MetO A*β* and 28.4 ± 0.7 in the WT, indicating that, on average, oxidation disrupts almost 6 (20%) intrapeptide contacts. When this calculation is restricted to long-range contacts occurring between amino acids separated by at least 5 residues, the corresponding values are $\langle {C}_{LR}\rangle =7.8\pm 0.6$ and 11.1 ± 0.7. This result suggests that 30% of long-range contacts are also destabilized by methionine oxidation, including the interactions between hydrophobic R2 and R4 regions (the number of R2–R4 contacts is reduced from 2.9 ± 0.3 to 0.6 ± 0.1). The reduction in A*β* intrapeptide interactions is visible in Fig. 3, which displays the difference contact map $\langle {\rm{\Delta }}C(i,j)\rangle$ for WT and MetO A*β*. The list of most affected contacts is presented in Table S2. Notably, 10 out of 15 such contacts are destabilized in MetO A*β*, supporting the analysis above. All five stabilized contacts occur between proximal residues ($|i-j|=2$ or 3) reflecting the formation of turns in MetO A*β*. The three long-range contacts in the table, all of which are destabilized, are Phe19–Ile31, the salt-bridge Asp23–Lys28, and Val24–Ile31. Their disruption implicates expansion of the A*β* structure as well as the loss of interactions between the two hydrophobic regions R2 and R4. Consistent with these observations, the radius of gyration of MetO A*β* $\langle {R}_{g}\rangle$ is 16.8 ± 0.2 Å, but is reduced to 14.8 ± 0.7 Å for the WT. Therefore, oxidation of Met35 causes the A*β* peptide to adopt more open, expanded conformations.

### A*β* interactions with DMPC bilayer

**Figure 4:**
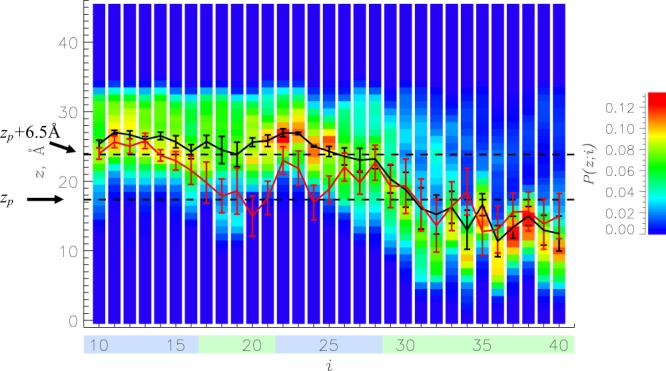
Color coded probabilities *P*(*z*; *i*) for MetO A*β* amino acids *i* to be at the distance *z* from the bilayer midplane. The average positions of MetO and WT A*β* amino acids, $\langle z(i)\rangle$, are represented by black and red lines. Vertical bars represent the standard error about the mean calculated from *n* = 5 trajectories. Two dashed lines separate the regions in which amino acids are considered inserted into the bilayer hydrophobic core, bound to the bilayer surface, or unbound (see Methods). An amino acid is bound to the bilayer if it is bound to its surface or inserted. Regions R1–R4 are colored according to Fig. 1a.

To explore MetO A*β* binding and insertion into the DMPC bilayer, we computed the probability *P*(*z*; *i*) for amino acid *i* to be at the distance *z* from the bilayer midplane (Fig. 4). Using *P*(*z*; *i*) it is straightforward to obtain the average position of amino acids along the z-axis, $\langle z(i)\rangle$. The figure shows that MetO A*β* peptide is divided into two almost equal regions - unbound N-terminus composed of amino acids 10 to 25 and bound C-terminus (26–40) (see Methods for definitions). In contrast, only a few N-terminal amino acids (10–13) in WT A*β* are classified as unbound with all others being bound to the DMPC bilayer. To supplement this conclusion, Table S3 presents the probabilities for A*β* sequence regions *k* = R1–R4 to be inserted *P*_*i*_(*k*), surface-bound *P*_*s*_(*k*), or unbound *P*_*u*_(*k*). We found that only one MetO A*β* region, the C-terminal R4 representing 39% of the sequence, is inserted into the bilayer (${P}_{i}(R4) > 0.5$) compared to two in WT A*β*, R2 and R4. More dramatic differences emerge if we compare unbound regions. Indeed, three MetO A*β* regions, R1–R3 representing 61% of the A*β* sequence, are classified as unbound from the bilayer, whereas there are no unbound regions for WT A*β*.

**Figure 5:**
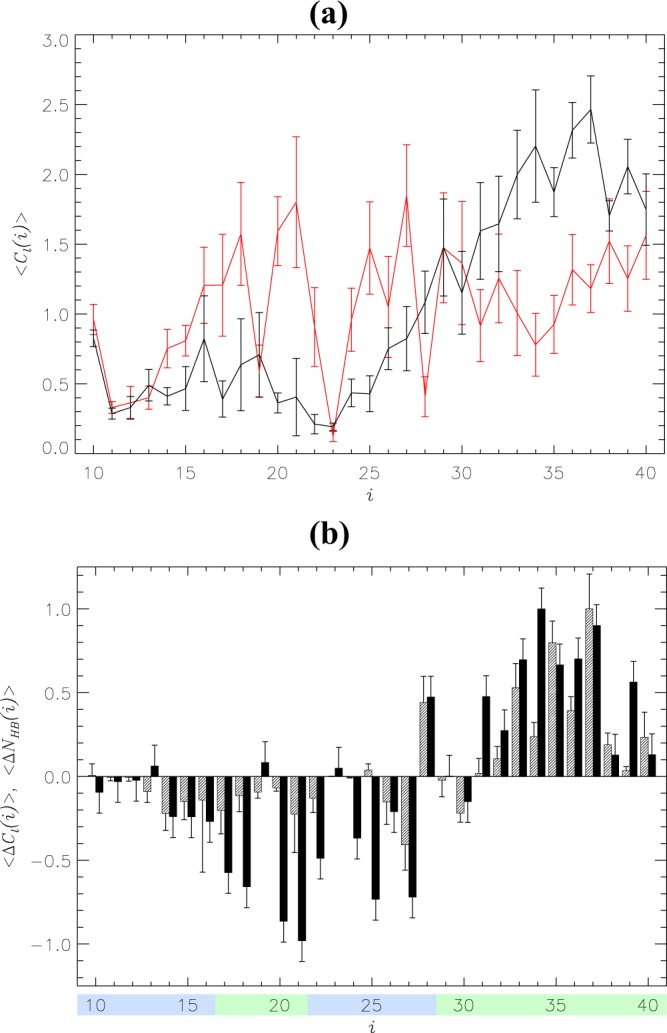
(**a**) The number of contacts $\langle {C}_{l}(i)\rangle$ formed between A*β* amino acids *i* and lipid groups. The data in black and red correspond to MetO and WT A*β*. (**b**) Black bars show residue specific changes in the number of binding contacts $\langle {\rm{\Delta }}{C}_{l}(i)\rangle =\langle {C}_{l}(i)\rangle -{\langle {C}_{l}(i)\rangle }_{WT}$, where $\langle {C}_{l}(i)\rangle$ and ${\langle {C}_{l}(i)\rangle }_{WT}$ are the numbers of contacts formed by MetO or WT amino acids, respectively. Residue specific changes in the number of hydrogen bonds $\langle {\rm{\Delta }}{N}_{HB}(i)\rangle$ (shaded bars) are defined similarly to $\langle {\rm{\Delta }}{C}_{l}(i)\rangle$. Vertical bars represent the standard error about the mean calculated from *n* = 5 trajectories. Regions R1–R4 are colored according to Fig. 1a. Both panels indicate that methionine oxidation strengthens the interactions between the A*β* C-terminus and the DMPC bilayer while weakening binding of other peptide regions.

The analysis above relies on the location of A*β* amino acids along the bilayer normal. To directly probe A*β* binding to the DMPC bilayer, we have examined the interactions between peptides and lipids. Although half of MetO A*β* amino acids are classified above as unbound, the peptide maintains its binding to the DMPC bilayer with the probability exceeding 0.99, i.e., on average, it keeps at least one contact with lipids. In all, upon binding, MetO A*β* forms $\langle {C}_{l}\rangle =32.3\pm 1.7$ contacts with lipid groups. Figure 5a shows that their distribution along the peptide sequence is remarkably skewed toward the C-terminus. Indeed, it follows from Fig. 5a that MetO A*β* C-terminal R4 forms 22.2 ± 3.0 contacts with lipids, whereas all other regions combined (R1–R3) establish only 10.1 ± 1.5 contacts. This result implies that 69% of MetO A*β* contacts with lipids are due to R4, which contains only 39% of all amino acids, whereas 31% of contacts are formed by R1–R3 regions representing 61% of the A*β* sequence. Although the total number of contacts between WT A*β* and the bilayer is approximately the same as for MetO A*β* ($\langle {C}_{l}\rangle =32.9\pm 6.9$), their distribution is notably different. In particular, the C-terminal R4 region of WT A*β* establishes only 14.6 ± 3.1 contacts with the bilayer (44% of all and 1.5-fold smaller than for MetO R4), whereas the rest of the peptide forms 18.4 ± 3.8 contacts (56%). Therefore, binding of the WT peptide implicates a more even distribution of binding interactions than in the oxidized peptide.

The residue specific differences in the number of binding contacts $\langle {\rm{\Delta }}{C}_{l}(i)\rangle$ formed by MetO and WT peptides are shown in Fig. 5b. This plot reveals that the C-terminal R4 is the only region that gains binding contacts, whereas all (with very few exceptions) amino acids from other three regions R1–R3 lose interactions with lipids. To illustrate this conclusion, we determined that the average value of $\langle {\rm{\Delta }}{C}_{l}(i)\rangle$ within R4 is 0.6, but it becomes negative (−0.4) within R1–R3. Similar implications follow from the analysis of hydrogen bonds between A*β* and the bilayer and the difference contact map $\langle {\rm{\Delta }}{C}_{l}(i,k)\rangle$, which distinguishes interactions with lipid groups *k* (see Supplementary Information). Indeed, Fig. 5b implicates remarkably non-uniform changes in hydrogen bonding along the A*β* sequence with the C-terminal R4 gaining hydrogen bonds and the other regions losing them. Taken together, our data suggest that methionine oxidation “polarizes” the A*β*-bilayer binding interface by strengthening binding of the A*β* C-terminus and weakening binding elsewhere. As a result, the C-terminus of MetO A*β* is embedded into the bilayer hydrophobic core with the rest of the peptide weakly coupled with the DMPC bilayer.

### Impact of A*β* on the bilayer structure

**Figure 6:**
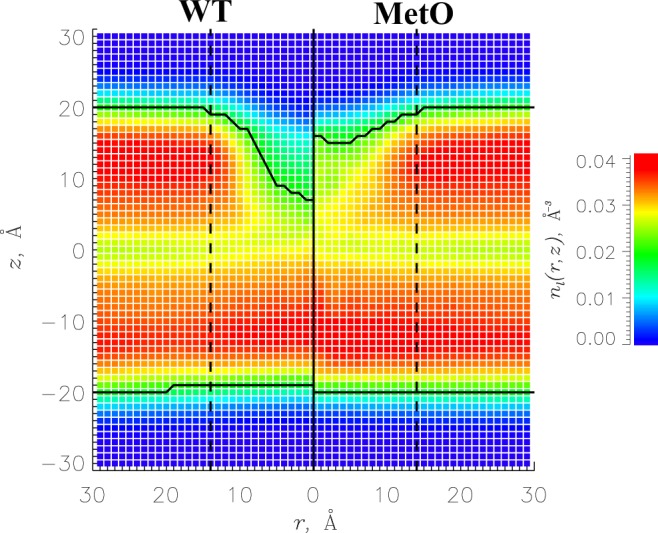
The number density *n*_*l*_(*r*, *z*) of lipid heavy atoms as a function of the distance *r* to the A*β* center of mass and distance *z* to the bilayer midplane. The left and right halves of the plot represent the indentation of the DMPC bilayer caused by WT and MetO A*β* peptides. Continuous black lines mark the bilayer boundaries. Vertical dashed lines separate proximal and distant regions. The panel demonstrates that binding of the MetO A*β* peptide causes considerably smaller thinning of the DMPC bilayer than of the WT.

Finally, we assess the structural perturbations caused within the bilayer by A*β* binding. In our previous work^30^, we concluded that WT A*β* indents the bilayer, creates a lipid density void beneath its binding footprint, and disorders proximal fatty acid tails. With MetO A*β* data available, we can determine if methionine oxidation augments these results. To begin, Fig. 6 presents the number density ${n}_{l}(r,z)$ of lipid heavy atoms as a function of the distance *r* to the A*β* center of mass and distance *z* to the bilayer midplane. The lipid density depression in the bilayer, which occurs upon WT A*β* binding, is largely subdued during MetO A*β* binding. We quantified this change using the following approach^45^. A bilayer is divided into proximal and distant regions depending on the distance to A*β*. The boundary between the two occurs at the distance *R*_*c*_ = 14 Å from the A*β* center of mass, where the number of A*β*-lipid contacts reaches maximum. In the distant region ($r > {R}_{c}$), we define the lipid-water boundary *z*_*b*_ from the condition ${n}_{l}(r > {R}_{c},{z}_{b})={n}_{w}({z}_{b})\equiv {n}_{b}$, where *n*_*w*_(*z*) is the water number density as a function of *z*. For both systems, *z*_*b*_ ≈ 20 Å. In the proximal region ($r < {R}_{c}$), we determine the boundary *z*_*b*_(*r*) setting $n(r,{z}_{b}(r))={n}_{b}$. The difference in *z*_*b*_(*r*) between the upper and lower leaflets produces the bilayer thickness *D*(*r*). The impact of A*β* can be assessed by computing the change in bilayer thickness ${\rm{\Delta }}D=D(r > 20\,\AA )-D(r < 6\,\AA )$. Then, the bilayer thinning for MetO and WT A*β* is ${\rm{\Delta }}D=4.5\pm 1.4$ and 12.7 ± 2.7 Å, respectively, indicating that the methionine-oxidized peptide produces a three-fold weaker impact on the bilayer thickness.

To evaluate the changes in bilayer density induced by A*β* binding we used two approaches. First, we determined variations in the lipid surface number density *n*_*s*_. In the distant region ${n}_{s}=0.016\pm 0.000\,{\AA }^{-2}$, which is reduced to $0.012\pm 0.000\,{\AA }^{-2}$ in the proximal region around bound MetO A*β*. Lipid density is further decreased to $0.010\pm 0.001\,{\AA }^{-2}$ in the center of the proximal region ($r < 6\,\AA$), thus representing a modest 38% density drop with respect to the distant value. For comparison, WT A*β* induces larger decrease in lipid density within the proximal region to $0.011\pm 0.002\,{\AA }^{-2}$, which becomes even more pronounced in its center, where ${n}_{s}=0.006\pm 0.003\,{\AA }^{-2}$ (a > 2.5-fold drop). Second, we considered variations in the volume number density of bilayer heavy atoms *n*_*l*_. In the distant bilayer region, *n*_*l*_ averaged over a leaflet ($0 < z < {z}_{b}$) is $0.035\pm 0.000\,{\AA }^{-3}$ and is reduced merely 20% to $0.028\pm 0.001\,{\AA }^{-3}$ in the proximal region around the bound MetO A*β* peptide. In contrast, WT A*β* causes a larger (31%) corresponding decrease in *n*_*l*_ to $0.024\pm 0.003\,{\AA }^{-3}$. Thus, both surface lipid and volume bilayer densities point to the same conclusion that oxidized A*β* peptide creates a smaller lipid density void within its binding footprint compared to the WT peptide. This outcome is consistent with a notably smaller thinning of the DMPC bilayer induced by MetO A*β* binding. The analysis of structural disordering in fatty acid tails due to A*β* binding is presented in the Supplementary Information.

## Discussion

Our isobaric-isothermal REST simulations demonstrate that adding a single oxygen atom to the A*β* monomer results in a dramatic structural reorganization in the bound peptide and DMPC bilayer. In particular, we observed almost complete disappearance of helical structure in the C-terminus of MetO A*β*. Indeed, according to Fig. 2a and Table S1, the C-terminal R4 helix fraction is reduced more than 5-fold. What is the mechanism responsible for destabilization of helical structure? In our previous study, we noted a large hydrophobic moment of the A*β* C-terminus, which matches those of other helix-forming peptides binding to cellular membranes^29^. Therefore, it is reasonable to suggest that, since oxidation changes the distribution of hydrophobicity along the C-terminus, its hydrophobic moment is affected. To check this conjecture, we constructed an idealized *α*-helix composed of all 12 C-terminal residues (Fig. S9) and computed its hydrophobic moment $\overrightarrow{\mu }$ as described in the Supplementary Information. The absolute value of $\overrightarrow{\mu }$ reflects the extent of partitioning of polar and apolar amino acids, whereas its direction points to the concentration of hydrophobic amino acids. Using the octanol-water hydrophobic scale from Wimley *et al*.^50^, we determined that *μ* for the WT A*β* C-terminus is 4.35 Å kcal/mol. Methionine oxidation adds a single polar atom (oxygen) to a hydrophobic C-terminus. Although, to our knowledge, the hydrophobicity for oxidized methionine has not been computed in the manner of the Wimley *et al*. scale^50^, the study of Black and Mould^51^ suggests that oxidized methionine is similar in its hydrophobicity to asparagine and glutamine. If these amino acids are substituted for Met35, the R4 hydrophobic moment decreases by 35 and 33% to 2.84 and 2.92 Å kcal/mol, respectively. A qualitatively similar observation follows if the Kyte-Doolite hydrophobicity scale^52^ is used. Therefore, addition of polar oxygen to Met35 decreases the C-terminus hydrophobic moment and disrupts its amphiphilic character. Figure 4 indicates that reduced Met35 in a helical state is inserted into the bilayer ($\langle z(i)\rangle  < {z}_{p}$). Accordingly, oxidation should destabilize the WT C-terminal helix at the bilayer hydrophobic/hydrophilic interface.

The aforementioned theoretical argument rationalizing helix disruption implies that orientations of amino acids in the MetO C-terminus, particularly of Met35, with respect to the bilayer normal must differ from those in the WT. To check this expectation, we computed in Fig. S10 the angles $\langle \theta (i)\rangle$ between the side chain vectors ${\overrightarrow{v}}_{i}$ and the reversed bilayer normal. (A vector ${\overrightarrow{v}}_{i}$ connects the C*α* atom and side chain center of mass of amino acid *i*). The figure reveals that $\langle \theta (i)\rangle$ for *i* = Met35 in the bound WT A*β* peptide is 32.6 ± 4.3°, whereas for MetO A*β* $\langle \theta (i)\rangle =114.4\pm 9.1^\circ$. Thus, oxidation of Met35 reorients its side chain from being directed toward the hydrophobic core of the bilayer to that pointing toward solvent. Indeed, according to Fig. 4 oxidized Met35 is located closer to the bilayer polar headgroup region than its reduced form. Met35 solvation can be directly measured if we compute the average number of water molecules within 6.5 Å of the Met35 side chain center of mass. For WT and MetO peptides, there are 3.4 ± 1.9 and 14.5 ± 0.4 water molecules hydrating Met35, respectively. Collectively, these results demonstrate that adding oxygen to Met35 significantly decreases the A*β* C-terminal hydrophobic moment, reorients and displaces Met35 toward solvent, and increases its solvation by more than four-fold.

Because methionine oxidation unravels the entire C-terminal helix, its effect extends over the C-terminus. Being constrained by the helical structure, two hydrophobic residues in the WT, *i* = Ala30 and Leu34, are directed toward solvent ($\langle \theta (i)\rangle  > 90^\circ$) and occur within the bilayer polar headgroup region (Fig. 4). In contrast, in MetO A*β* both amino acids are pointed toward the bilayer midplane (Fig. S10) and Leu34 is inserted into the hydrophobic core. As a result, in the MetO peptide, which is no longer constrained by the C-terminal helix, the sole R4 amino acid oriented toward solvent is Met35. Furthermore, oxidation of Met35 increases total A*β* hydration. Specifically, 228 ± 27 water molecules are bound, on average, to the WT peptide, whereas for MetO A*β* their number increases to 276 ± 8. Unraveling of C-terminal helix also disrupts intrapeptide contacts, as MetO A*β* sheds 2.3 short-range and 3.4 long-range contacts compared to WT A*β*. Loss of intrapeptide contacts further destabilizes helical structure. Taken together, these findings demonstrate that the mechanism of C-terminal helix unraveling can be ultimately traced to considerable decrease in the C-terminus hydrophobic moment in the oxidized peptide.

Based on the analysis of bilayer density, thinning, and distribution of MetO A*β* amino acids along the bilayer normal, we concluded that MetO A*β* amino acids tend to mix with lipids rather than form a peptide-only phase within the bilayer. What is then the reason for better “mixing” propensity of the oxidized peptide? To answer this, we probed the fluctuations in A*β* structure by computing the standard deviations in the peptide $$\varphi $$ and $$\psi $$ dihedral angles^53^. Figure S11 presents the difference in these dihedral angle fluctuations, $${\rm{\Delta }}\delta \varphi (i)$$ and $${\rm{\Delta }}\delta \psi (i)$$, for all amino acids *i* in MetO and WT A*β* (see Fig. S11 caption for definitions). The figure shows that 23 out of 29 amino acids (excluding the termini) have positive $${\rm{\Delta }}\delta \varphi (i)$$ and/or $${\rm{\Delta }}\delta \psi (i)$$ implicating enhanced flexibility of the oxidized peptide. To evaluate the bias in the distributions of $${\rm{\Delta }}\delta \varphi (i)$$ and $${\rm{\Delta }}\delta \psi (i)$$, we computed the ratios $${f}_{\varphi }={\sum }_{+}\,{\rm{\Delta }}\delta \varphi (i)/{\sum }_{-}\,-{\rm{\Delta }}\delta \varphi (i)$$ and $${f}_{\psi }={\sum }_{+}\,{\rm{\Delta }}\delta \psi (i)/$$$${\sum }_{-}\,-{\rm{\Delta }}\delta \psi (i)$$, where the subscripts + and − refer to summation over positive or negative $${\rm{\Delta }}\delta \varphi (i)$$ and $${\rm{\Delta }}\delta \psi (i)$$. We found that $${f}_{\varphi }=2.8 > 1$$ and $${f}_{\psi }=5.2 > 1$$ indicating that oxidation causes a net increase in the fluctuations in A*β* structure. Calculating $${f}_{\varphi }$$ and $${f}_{\psi }$$ for the C-terminus only, we find 117.4 and 4.8 suggesting that this A*β* region experiences particularly enhanced fluctuations.

In summary, these results argue that, consistent with the net loss of intrapeptide contacts, unraveling of C-terminal helix, and overall peptide expansion, MetO A*β* is more flexible than the WT. More open MetO A*β* conformations subject to large fluctuations afford better mixing with lipid atoms manifested in almost two-fold higher surface number density of lipids within the proximal region center and 1.5-fold higher number of A*β*-lipid contacts in the C-terminus compared to the WT peptide. Furthermore, though weak bilayer thinning is expected to reduce lipid structural perturbation, better mixing of lipids with amino acids evidently causes a marginal net increase in lipid structure disordering as noted in the Supplementary Information.

Finally, given considerable changes occurring in the mechanism of A*β* binding in response to methionine oxidation, it is likely that oxidized and reduced peptides bind to the DMPC bilayer with different affinities. To investigate this possibility, we computed the free energy of binding using replica exchange conformational sampling and the MM-GBSA approach^54^. The free energy *G* of a system can be decomposed as

1 $$G={E}_{mm}+{G}_{solv,p}+{G}_{solv,ap}-TS,$$

where *E*_*mm*_ is the molecular mechanical energy, *G*_*solv*,*p*_ is the polar contribution to the solvation free energy computed using the Generalized Born implicit solvent model^55^ with the dielectric constant of 78.5, *G*_*solv*,*ap*_ is the apolar contribution to the solvation free energy estimated from the solvent accessible surface area with a 1.4 Å probe radius and nonpolar surface tension coefficient *γ* = 0.005 kcal/mol/Å^2^, and *TS* is the conformational entropic term. The latter was computed using Gibbs formula for entropy (see Methods). Taking into account that our simulation system contains two non-interacting A*β* peptides, the free energy of A*β* binding to the DMPC bilayer is

2 $${\rm{\Delta }}{G}_{b}=({G}_{A\beta +DMPC}-2{G}_{A\beta }-{G}_{DMPC})/2,$$

where ${G}_{A\beta +DMPC}$, *G*_*Aβ*_, and *G*_*DMPC*_ are the free energies of the DMPC bilayer with two bound A*β* peptides, of the unbound peptide in water, and peptide-free DMPC bilayer. Then, the change in the binding free energy between MetO and WT A*β* peptides is

3 $${\rm{\Delta }}{\rm{\Delta }}{G}_{b}=({G}_{MetOA\beta +DMPC}-{G}_{WTA\beta +DMPC}-2\ast ({G}_{MetOA\beta }-{G}_{WTA\beta }))/2.$$

In Eq. (3), the free energy of the peptide-free DMPC bilayer cancels out and each remaining term is computed using Eq. (1). REST simulations of MetO A*β* bound to the DMPC bilayer were used to determine ${G}_{MetOA\beta +DMPC}$, whereas our previous REMD/REST simulations of WT A*β* in water and bound to the DMPC bilayer were utilized to compute *G*_*WTAβ*_ and ${G}_{WTA\beta +DMPC}$^29,46,56^. Additional REST simulations of oxidized A*β*10–40 monomer in water were performed to estimate *G*_*MetOAβ*_ (see Methods). Detailed account of binding energetics is given in the Supplementary Information.

According to Eq. (3) and Table S5 ${\rm{\Delta }}{\rm{\Delta }}{G}_{b}=17\pm 9$ kcal/mol implying that MetO A*β* has lower binding affinity than the WT. It is instructive to decompose ${\rm{\Delta }}{\rm{\Delta }}{G}_{b}={\rm{\Delta }}{\rm{\Delta }}{E}_{mm}+{\rm{\Delta }}{\rm{\Delta }}{G}_{solv,p}+{\rm{\Delta }}{\rm{\Delta }}{G}_{solv,ap}-T{\rm{\Delta }}{\rm{\Delta }}S$. It follows from Table S5 that the difference in molecular mechanical energy ${\rm{\Delta }}{\rm{\Delta }}{E}_{mm}=70\pm 18$ kcal/mol is unfavorable, being partially compensated by the polar contribution to solvation ${\rm{\Delta }}{\rm{\Delta }}{G}_{solv,p}=-54\pm 23$ kcal/mol. Furthermore, the difference in apolar contribution to solvation ${\rm{\Delta }}{\rm{\Delta }}{G}_{solv,ap}=2\pm 1$ kcal/mol is minor, whereas the entropic contribution ${\textstyle \text{-}}\,T{\rm{\Delta }}{\rm{\Delta }}S\approx 0\pm 0$ kcal/mol is negligible. Thus, oxidation reduces the binding affinity of A*β* to the DMPC bilayer through the interplay of two opposing factors. The one disfavoring binding of oxidized A*β* to the DMPC bilayer is the loss of favorable intrapeptide interactions upon binding, whereas the factor favoring binding is better hydration of the bound oxidized peptide.

It is important to compare our findings with previous studies. Constant temperature molecular dynamics^24,57^, replica exchange molecular dynamics^23^, and NMR spectroscopy^22^ have been used to study the effects of methionine oxidation on A*β*1–40 and A*β*1–42 peptides in water. The consensus emerging from these studies is that oxidation has a destabilizing effect on the peptide structure. For example, methionine oxidation in A*β*1–42 monomers weakens the long range intrapeptide contacts^23^ and increases the accessible surface area of methionine by more than two-fold^24^. Another molecular dynamics study has reported a decrease in *β* structure content within the C-terminus of A*β*1–40 monomers^57^, whereas NMR data suggest enhanced fluctuations in the A*β*1–42 C-terminus caused by oxidation^22^. It is notable that, despite the difference in local environment (water vs lipid bilayer), these outcomes qualitatively agree with the observations drawn from our study. More relevant to our findings are the structures of WT and MetO A*β*1–40 in a water-SDS micelle environment determined by NMR spectroscopy (PDB IDs 1BA4 and 1BA6)^12,14^. To investigate their secondary structure propensities we applied STRIDE^48^. Because each PDB entry contains 10 conformers, we assumed that a residue is in a helical state if that state is present in more than half of all conformers. In WT A*β*, helix spans the residues Gln15–Gly37; however, for MetO A*β*, helix is contracted to the sequence interval Lys16–Ser26, i.e., the C-terminal portion of the WT helix, Asn27–Gly37, is absent. The disappearance of helix in the C-terminus of the oxidized peptide is consistent with our REST simulations, which reported seven residues forming helix in the WT A*β* C-terminus and none in the oxidized form. Several experimental studies^13,14,17,18^ have noted that delayed aggregation of MetO A*β* peptides is responsible for a less severe cytotoxic effect. We speculate that our central result concerning reduced affinity of binding of oxidized A*β* peptide to the lipid bilayer constitutes a molecular basis for this experimental observation. We postulate that reduced affinity of MetO A*β* to the DMPC bilayer vs the WT could play a role in slowing down the MetO A*β* aggregation offsetting its cytotoxic effects. It is also worth noting that the value of ${\rm{\Delta }}{\rm{\Delta }}{G}_{b}=17$ kcal/mol is consistent with the experimental magnitude of free energies of peptide binding to lipid bilayers^58^.

## Conclusions

Using all-atom explicit solvent replica exchange molecular dynamics simulations with solute tempering, we showed that methionine oxidation changes the mechanism of A*β*10–40 peptide binding to the DMPC bilayer. By comparing binding of oxidized and reduced peptides, five consequences of this common post-translational modification were identified. First, Met35 oxidation changes secondary structure of the bound A*β* peptide by unraveling C-terminal helix and promoting turn conformations. Second and related to the first, oxidation destabilizes intrapeptide interactions and causes expansion of the bound peptide. These two outcomes are explained by the loss of amphiphilic character of the C-terminal helix in the oxidized peptide quantified by a reduced hydrophobic moment. Third, oxidation “polarizes” A*β* binding to the DMPC bilayer by strengthening the interactions of the C-terminus with lipids, but largely releasing the rest of the peptide from the bilayer surface. Fourth, compared to the wild-type, binding of the oxidized peptide induces significantly smaller thinning of the bilayer and causes relatively minor drop in lipid density within the binding footprint. These observations were explained by mixing of oxidized peptide amino acids with lipids promoted by enhanced A*β* conformational fluctuations. Fifth and most important, methionine oxidation reduces the affinity of A*β* binding to the DMPC bilayer mainly by disrupting favorable intrapeptide interactions upon binding, which offset the energetic gains from better hydration. We propose that reduced binding affinity of the oxidized A*β* peptide serves as a molecular basis for its reduced cytotoxicity.

## Supplementary information

Supplementary Information

## Data Availability

The datasets generated and analysed during the current study are available from the corresponding author on reasonable request.
