# Supplementary material for: Methionine Oxidation Changes the Mechanism of Aβ Peptide Binding to the DMPC Bilayer
Source: Sci Rep. 2019 Apr 11;9:5947. doi: 10.1038/s41598-019-42304-9 (PMC6459879; doi:10.1038/s41598-019-42304-9)
Supplement: Supplementary file 1 — Supplementary Information [file 41598_2019_42304_MOESM1_ESM.pdf]

Methionine Oxidation Changes the Mechanism of Aβ Peptide Binding to the DMPC Bilayer

Christopher Lockhart, Amy K. Smith, and Dmitri K. Klimov
School of Systems Biology, George Mason University, Manassas, VA 20110
E-mail: dklimov@gmu.edu

Supplementary Information

We performed isobaric-isothermal replica exchange molecular dynamics simulations with solute tempering (REST) of methionine oxidized Aβ10-40 monomers binding to the DMPC bilayer. In our simulations we used all-atom explicit solvent model. Following REST formalism, we introduced R=8 replicas distributed in the temperature interval from T_0=330K to T_{R-1}=430 K. It is important to verify the performance of REST algorithm and convergence of conformational sampling.

Enthalpic distributions: REST scales solvent interactions largely eliminating their contribution to the exchange probability ω, while leaving solute contributions intact [1,2]. Because solute represents a small fraction of all atoms, the enthalpic terms contributing to ω have broad distributions allowing one to reduce the number of replicas without sacrificing the overlaps of their enthalpies. From the perspective of full REST enthalpies H_r of replicas r application of scaling factors effectively keeps the solvent cold at temperature T_0 thus bringing the distributions of H_r closer to each other. To verify that this is indeed the case we plot in Fig. S1 the probability distributions $P(\beta_r H_r,T_r)$ of enthalpies H_r. Excellent overlaps of their distributions indicate that REST performs as expected.

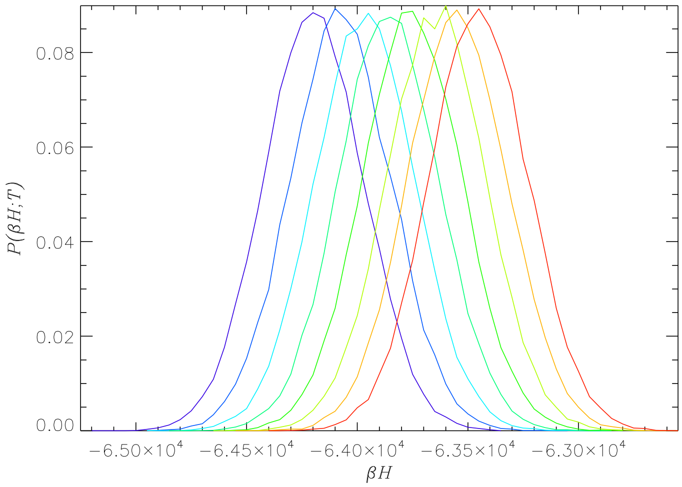

Fig. S1 Probability distributions $P(\beta_r H_r,T_r)$ for enthalpies H_r. Significant overlap between adjacent replica distributions is a prerequisite for efficient mixing of replicas over temperature scale.

Sampling convergence: To assess the convergence of REST simulations, we present in Fig. S2 the number of unique states N_s as a function of REST equilibrium simulation time τ_{sim}. A state (H,X) is defined by the enthalpy H, in which the bias introduced by REST is removed, and the structural probe X, where X = C, the number of intrapeptide contacts, or

C_l, the number of peptide-lipid contacts. Fig. S2 demonstrates that, within the timescale of our simulations, both N_s approximately level off indicating an exhaustion of new states. It is important to emphasize that saturation of new states constitutes a necessary condition for sampling convergence.

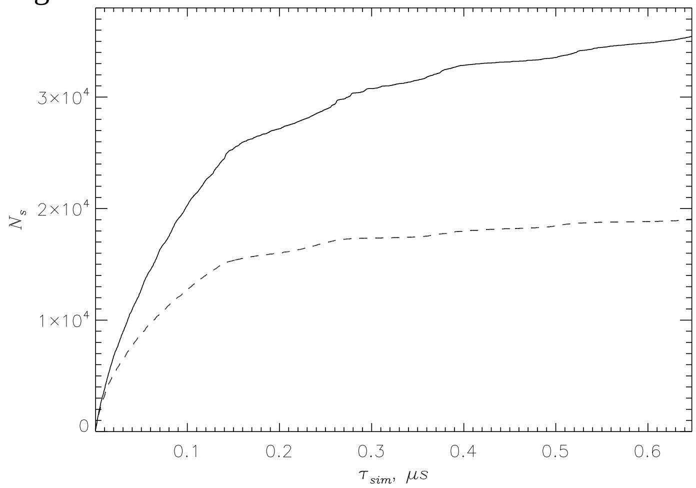

Fig. S2 The number of unique states N_s acquired during equilibrium simulation time τ_{sim}. The dashed line corresponds to unique states (H,C), whereas the solid black line corresponds to the states (H,C_l). Gradual saturation of N_s indicates that unique states become approximately exhausted.

The R=8 replicas in our REST simulations should exhibit a random walk over REST temperatures. To probe this expectation, we display in Fig. S3 the distribution of replicas at each REST iteration. Importantly, this figure presents a color mosaic, indicating efficient replica mixing across temperatures. Quantitatively, replica mixing can be assessed by computing the tunneling time τ_R, the average time required for a replica to transit from the lowest to highest REST temperatures (or in reverse) [3]. Average τ_R for all five REST trajectories is 3.2 ns, i.e., within a single REST trajectory a replica makes approximately three complete round trips through the REST temperature range.

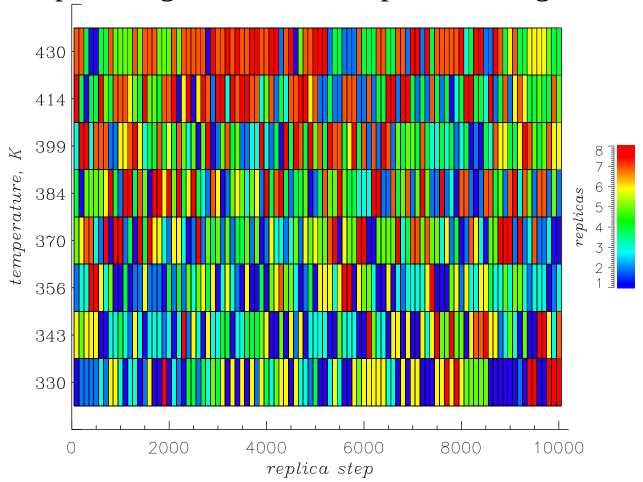

Fig. S3 Random walk of replicas over temperatures in a typical REST trajectory. The colors in the right scale represent the distribution of replicas over temperatures at the beginning of the trajectory. Production of a color mosaic indicates efficient replica mixing.

Replica mixing can be further quantified following Han and Hansmann [4]. Specifically, they defined the parameter $m(T)$, which tracks the amount of simulation time t_r a replica r spends at temperature T, as

$$m(T) = 1 - \frac{\sqrt{\sum_{r=1}^{R} t_r^2}}{\sum_{r=1}^{R} t_r} \quad \text{(Eq. S1)}$$

If all R=8 replicas are uniformly distributed across temperatures, $m(T)=1-1/R^{1/2}\approx 0.65$. In other words, $m(T)\approx 0.65$ is the optimum theoretical value indicating ideal replica mixing. Fig. S4 demonstrates that this is approximately true for our simulation system.

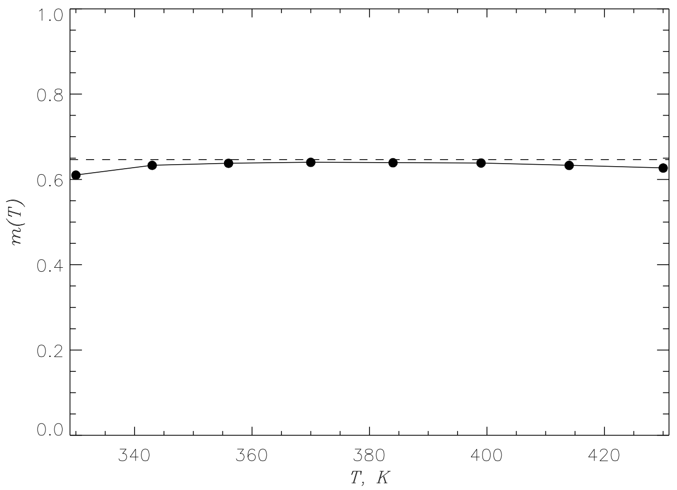

Fig. S4 The mixing parameter $m(T)$ as a function of the REST temperature T. The optimum theoretical value of 0.65 is marked by a dashed line.

Next, we examined the relaxation of energetic and structural quantities to their baseline equilibrium values. Specifically, we checked the enthalpy of the entire system H as a function of REST iteration at 330K and found that it reaches the baseline in each trajectory within the time interval not exceeding 3.8 ns. As a further test we considered the location of Aβ peptide in the bilayer. To this end, Fig. S5 presents the positions of Aβ and its C-terminus centers of mass Z_{COM} as a function of REST iteration. This figure suggests that the peptide position along the bilayer normal is well equilibrated at 330K.

Because REST strengthens solvent interactions in replicas $r > 0$, it is important to check that REST and traditional replica exchange molecular dynamics (REMD) produce consistent results. In our previous study [2] we have performed this test for the system of WT Aβ10-40 peptide binding to the DMPC lipid bilayer. Specifically, the averages and the distributions of quantities probing peptide and bilayer structure were in excellent agreement, including intrapeptide contact maps, distributions of amino acids along the bilayer normal, bilayer density profiles, and S_{CD} distributions. Therefore, we believe that REST collects unbiased conformational sampling.

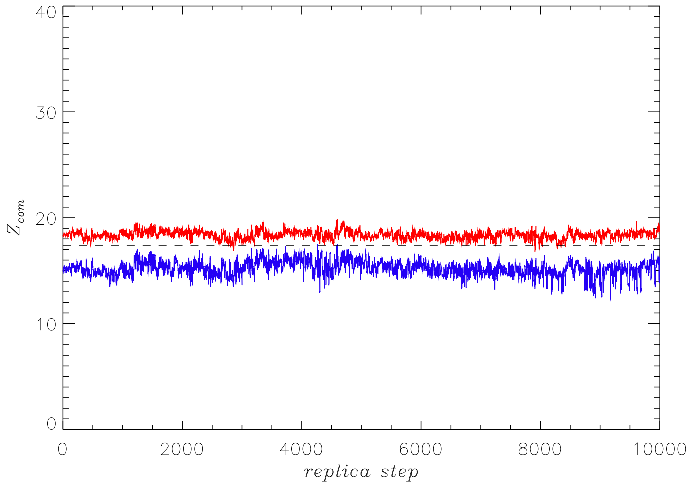

Fig. S5 The positions of the centers of mass Z_{COM} of Aβ (in red) and its C-terminus (in blue) as a function of REST iteration. The lack of drift in these quantities suggests system equilibration. Dashed line indicates the position of the center of mass of phosphorous atoms in a leaflet.

Finally, we discuss the role of initial structures in REST simulations. To prepare unique starting structures for each trajectory, we placed peptides in random orientation near the DMPC bilayer and performed short simulations until the peptides became bound to the bilayer (Fig. S6). These bound structures were then utilized to initiate REST. The average RMSD between these initial structures was 40.1 Å (or 44.8 Å for WT Aβ simulations) suggesting that they are conformationally dissimilar. Protocol for WT Aβ simulations is described elsewhere [2].

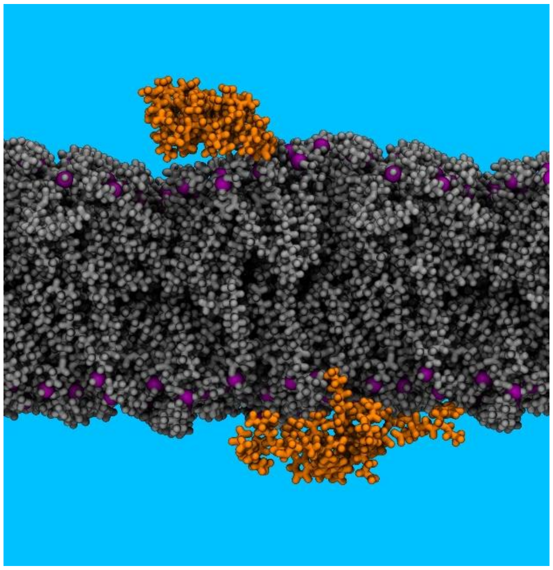

Fig. S6 One of initial structures for REST simulations. The DMPC lipids are in grey except for phosphorous atoms shown in purple. MetO Aβ peptides are in orange, whereas water is presented in blue. Ions are omitted.

Aβ secondary structure: To facilitate the analysis of Aβ secondary structure, we present in Table S1 the helical <*H(k)*>, turn <*T(k)*>, or random coil <*RC(k)*> fractions within the sequence regions *k*=R1-R4 of MetO and WT Aβ. The table reveals that in WT the only sequence region forming stable (fraction > 0.5) secondary structure is the C-terminal R4

featuring helical state. In contrast, no MetO Aβ regions adopt helical conformation, whereas three regions (R2-R4) are populated by stable turn structure.

Table S1 Secondary structure propensities in Aβ sequence regions R1-R4[a,b]

System	Quantity	R1	R2	R3	R4
**MetO Aβ**	***<H>***	0.39±0.09	0.21±0.05	0.20±0.04	0.12±0.03
	<T>	0.36±0.05	0.60±0.04	0.60±0.04	0.55±0.03
	<RC>	0.25±0.07	0.19±0.02	0.19±0.02	0.32±0.02
**WT Aβ**	***<H>***	0.14±0.08	0.10±0.03	0.39±0.05	0.65±0.05
	<T>	0.46±0.06	0.39±0.03	0.41±0.06	0.10±0.05
	<RC>	0.40±0.04	0.50±0.01	0.20±0.02	0.26±0.01

[a] Highlighted cells mark the regions with stable secondary structure.
[b] Uncertainties are standard errors about the mean calculated from n=5 trajectories.

Aβ tertiary structure: To assess Aβ tertiary structure we use the difference contact map $<\Delta C(i,j)> = <C(i,j)> - <C(i,j)>_{WT}$, where $<C(i,j)>$ and $<C(i,j)>_{WT}$ are the contact maps for MetO and WT Aβ peptides, respectively, and i and j refer to amino acids. Table S2 lists the contacts most affected by oxidation ($|<\Delta C(i,j)>| \geq 0.35$). Two-third (10 out 15) of such contacts are destabilized by oxidation. In particular, all long-range ($|i-j| \geq 5$) contacts and the majority (58%) of short-range ($|i-j| < 5$) contacts are weakened by oxidation.

Table S2 Intrapeptide contacts most affected by methionine oxidation[a]

Rank	*i*	*j*	$<\Delta C(i,j)>$
1	33	36	-0.81
2	33	37	-0.78
3	34	36	0.62
4	32	35	-0.52
5	19	31	-0.46
6	35	38	0.44
7	23	28	-0.43
8	24	31	-0.41
9	33	35	0.40
10	32	34	0.40
11	27	30	0.38
12	29	32	-0.37
13	31	34	-0.36
14	17	19	-0.36
15	26	28	-0.35

[a]Highlighted cells indicate long-range intrapeptide contacts.

Aβ-bilayer interactions: Our simulations probe the equilibrium binding of the MetO Aβ peptide to the DMPC bilayer. In Table S3, we present the probabilities for Aβ sequence regions k=R1-R4 to be localized at the bilayer surface $P_s(k)$, inserted into the bilayer hydrophobic core below lipid phosphorus atoms $P_i(k)$, or unbound $P_u(k)$. The definitions of these probabilities and their analysis are given in the main text.

Table S3 Probabilities for Aβ sequence regions R1-R4 to occur along the bilayer normal[a,b]

System	Quantity	R1	R2	R3	R4
**MetO Aβ**	P_s	0.37±0.02	0.40±0.04	0.42±0.05	0.19±0.03
	P_i	0.03±0.02	0.06±0.06	0.06±0.02	0.69±0.13
	P_u	0.60±0.04	0.54±0.09	0.52±0.07	0.12±0.10
**WT Aβ**	P_s	0.45±0.06	0.28±0.07	0.46±0.05	0.12±0.04
	P_i	0.09±0.03	0.52±0.18	0.30±0.10	0.66±0.19
	P_u	0.46±0.08	0.20±0.12	0.24±0.14	0.22±0.15

[a] Highlighted cells mark the probabilities greater than 0.5.
[b] Uncertainties are standard errors about the mean calculated from n=5 trajectories.

To supplement the analysis of binding interactions between Aβ peptide and DMPC bilayer, we considered the formation of hydrogen bonds. One may conjecture that unraveling of the C-terminal helix makes hydrogen donors and acceptors in Aβ backbone available for interactions with DMPC lipids. To assess this possibility, we calculated the numbers of hydrogen bonds $<N_{HB}(i)>$ between MetO Aβ amino acids *i* and lipids and then determined their difference with respect to the WT peptide, $<\Delta N_{HB}(i)>$ (Fig. 5b). Note that DMPC lipids can only serve as hydrogen acceptors (Fig. 1c), whereas hydrogen donors must come from Aβ. Furthermore, in the WT and MetO Aβ C-termini, hydrogen donors are only available in the peptide backbone. In total, there are $<N_{HB}>$=4.5±0.3 hydrogen bonds between MetO Aβ and lipids compared to 3.5±0.9 formed by the WT implying that one additional hydrogen bond is established overall between MetO Aβ and the bilayer. Importantly, Fig. 5b implicates remarkably non-uniform changes in hydrogen bonding along Aβ sequence with the C-terminal R4 region gaining hydrogen bonds and the other regions losing them. Indeed, the MetO C-terminus forms approximately two additional hydrogen bonds with the bilayer, whereas about one less bond occurs between MetO regions R1-R3 and the bilayer. These computations support the assumption made above that helix unraveling in the MetO C-terminus makes the peptide backbone available for hydrogen bonding with the bilayer.

We further analyze Aβ-lipid interactions by computing the difference $<\Delta C_l(i,k)>$ in the number of contacts formed between amino acids *i* and lipid structural groups *k*=G1-G5 caused by oxidation. Specifically, $<\Delta C_l(i,k)>=<C_l(i,k)> - <C_l(i,k)>_{WT}$, where $<C_l(i,k)>$ and $<C_l(i,k)>_{WT}$ are the peptide-lipid contact maps reporting the number of contacts between *i* and *k* for MetO and WT Aβ, respectively. The difference contact map $<\Delta C_l(i,k)>$ is presented in Fig. S7, whereas Table S4 lists the contacts most affected by oxidation ($|<\Delta C_l(i,k)>| \geq 0.35$). Consistent with Fig. 5b, this figure demonstrates that the interactions between the MetO Aβ C-terminus R4 and all lipid structural groups *k*=G1-G5 are generally enhanced. This outcome is contrasted with elsewhere in the sequence, which features mostly a loss in Aβ-lipid contacts due to methionine oxidation. Interestingly, the interaction most strengthened by oxidation as reflected in the largest positive $<\Delta C_l(i,k)>$ in Table S4 occurs between oxidized Met35 and the lipid phosphate group G2. In WT Aβ, the number of contacts between Met35 and G2 is 0.02, but it increases to 0.83 upon oxidation becoming the most stable peptide-lipid group interaction. The next two interactions enhanced by oxidation in descending order are those between G1 and Gly33 or Met35 (Table S4). Aβ-lipid interactions most destabilized by oxidation are the contacts

between Phe20 and fatty acid tails G4 and G5. These changes reflect the expulsion of MetO Aβ R2 region from the bilayer. Importantly, out of 12 contacts with positive <$\Delta C_l(i,k)$> in Table S4 11 are associated with the C-terminal R4 region. These results are consistent with the enhanced binding interactions established between MetO Aβ C-terminus and the bilayer. Interestingly, stronger binding interactions are particularly evident for choline (G1), phosphorous (G2), and glycerol (G3) groups, which contribute 82% of the most affected contacts in Table S4.

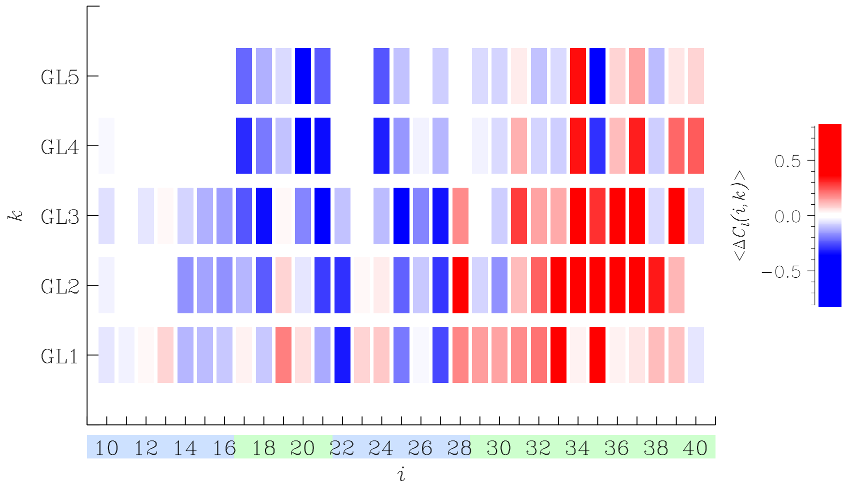

Fig. S7 Difference contact map <$\Delta C_l(i,k)$> reporting the change in the number of contacts formed between amino acids *i* and lipid structural groups *k*=G1-G5 caused by oxidation. Regions R1-R4 are colored according to Fig. 1a.

Table S4 Aβ-lipid contacts most affected by methionine oxidation

Rank	*i*	*K*	<$\Delta C_l(i,k)$>
1	35	G2	0.81
2	33	G1	0.56
3	20	G5	-0.54
4	20	G4	-0.53
5	35	G1	0.53
6	21	G3	-0.45
7	33	G2	0.43
8	37	G2	0.41
9	36	G2	0.41
10	36	G3	0.40
11	25	G3	-0.40
12	34	G3	0.40
13	35	G5	-0.40
14	37	G3	0.39
15	39	G3	0.35
16	34	G2	0.35
17	28	G2	0.35

Impact on bilayer structure: We examined the disordering in lipid structure produced by MetO and WT Aβ peptides. Fig. S8a presents the lipid carbon-deuterium order parameter -<$S_{CD}(i)$> computed for each carbon *i* in *sn-2* fatty acid tails from the bilayers featuring bound MetO and WT Aβ. In the distant regions of both bilayers the average -<S_{CD}> is 0.16±0.00. For proximal lipids surrounding MetO Aβ, average -<S_{CD}> is 0.12±0.00 and further decreases to 0.10±0.01 in the center of proximal region (*r*<6Å). For comparison, the corresponding values for the WT Aβ bilayer are 0.13±0.00 and 0.11±0.02 implying that the difference between MetO and WT -<S_{CD}> values is marginal. Another quantity probing fatty acid tail disordering is a tilt angle γ (see Methods). From its probability distributions shown in Fig. S8b, we found that <γ> averaged over distant lipids is 149±0°. In the proximal region around the MetO peptide, the tilt angle is marginally smaller than near WT Aβ (144±1° vs 145±0°, respectively). Similar result follows if we consider the centers of proximal regions near MetO and WT peptides (140±1° against 141±3°). Thus, the oxidized Aβ peptide induces marginally stronger structural perturbation in lipid fatty acid tails compared to the WT. This finding appears at variance with the much weaker impact of MetO Aβ binding on the DMPC bilayer structure compared to its WT counterpart displayed in Fig. 6. In Discussion we rationalize these conflicting observations.

(a)

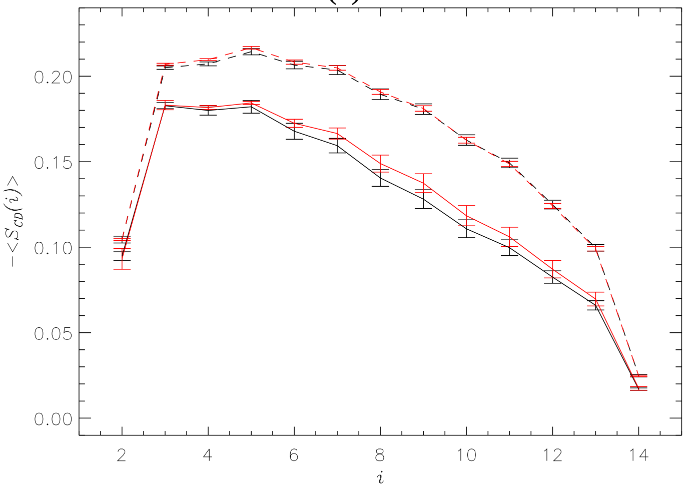

(b)

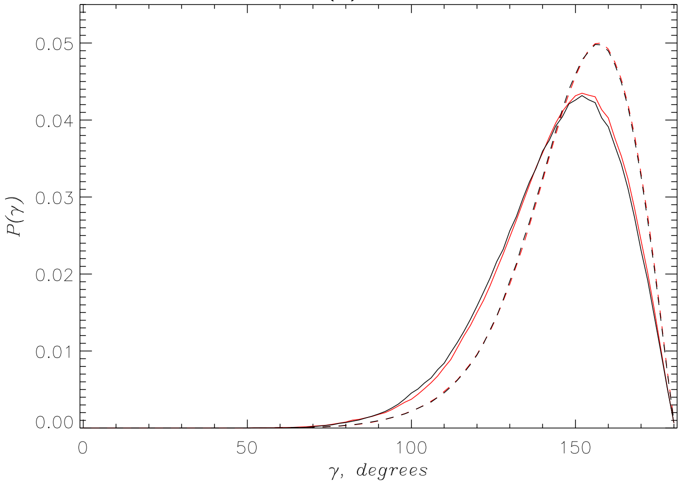

Fig. S8 (a) The lipid carbon-deuterium order parameter -<$S_{CD}(i)$> computed for each carbon *i* in *sn-2* fatty acid tails. Vertical bars represent the standard error about the mean calculated from n=5 trajectories. (b) Probability distributions $P(\gamma)$ for lipid *sn-2* fatty acid tails to be tilted at an angle γ from the bilayer normal. In both panels data for MetO and WT Aβ are in black and red, respectively. The solid and dashed lines refer to the proximal and distant lipids. The panel reveals that binding of MetO Aβ results in marginally stronger structural disordering of the DMPC fatty acid tails than of the WT.

Computation of hydrophobic moment: To compute hydrophobic moment, we created an idealized α-helix formed by all 12 C-terminal Aβ residues. To this end, the positions of Cα atoms of amino acids were placed on the circle with the 1 Å radius. Each successive Cα atom is rotated δ=100° relative to the previous one. Following [5] a hydrophobic moment is $\vec{\mu} = \mu_x\vec{\imath} + \mu_y\vec{\jmath}$, where $\mu_x = \sum_{k=0}^{11} H_k\cos(\delta k)$, $\mu_y = \sum_{k=0}^{11} H_k\sin(\delta k)$, H_k is a hydrophobicity of amino acid k [6], and $\vec{\imath}$ and $\vec{\jmath}$ are unit vectors. Their initial orientation is arbitrary and was chosen to direct vector $\vec{\mu}$ as in Fig. S9.

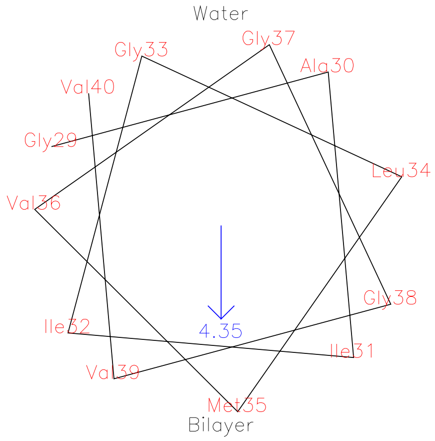

Fig. S9 Helix wheel representing angular distribution of amino acids in an ideal C-terminal α-helix of the WT Aβ peptide. Blue arrow shows hydrophobic moment vector $\vec{\mu}$. Taking into account the positions of individual WT amino acids along the bilayer normal (Fig. 4), we approximately identify the sides of helix wheel, which are oriented toward solvent and bilayer.

Side chain orientation: To probe the orientation of Aβ side chains *i* we compute in Fig. S10 the average angles <$\theta(i)$> formed between side chain vectors $\vec{v_i}$ and the reversed bilayer normal (see figure caption). As discussed in the main text, this plot shows that Met35 changes orientation when oxidized and points toward solvent instead of the bilayer midplane.

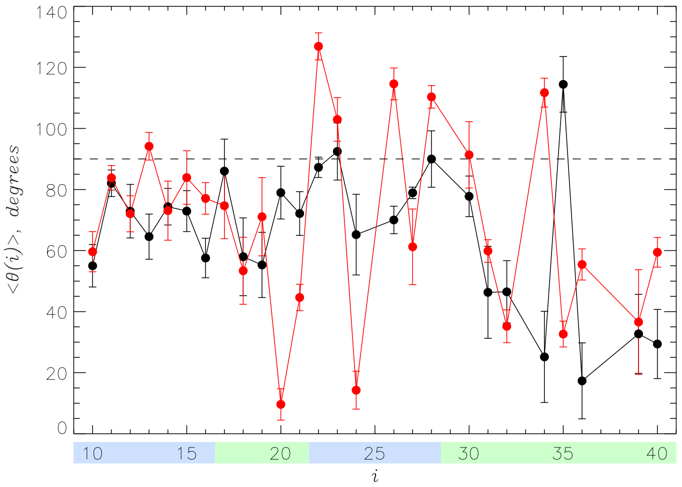

Fig. S10 Distribution of angles <*θ(i)*> between amino acid side chains and the reversed bilayer normal. Glycines were excluded from the analysis. The data in black and red correspond to MetO and WT Aβ, respectively. An angle of 0° indicates that a side chain points directly toward the bilayer midplane. Regions R1-R4 are colored according to Fig. 1a. Vertical bars represent the standard error about the mean calculated from n=5 trajectories.

Fluctuations in Aβ structure: To assess rigidity of Aβ structures, we computed the standard deviations in peptide backbone dihedral angles δ*φ(i)* and δ*ψ(i)* for each amino acid *i*. Fig. S11 plots the differences in the dihedral angle standard deviations, Δδ*φ*(*i*) and Δδ*ψ*(*i*), between MetO and WT Aβ. Visual inspection suggests that MetO Aβ experiences higher fluctuations than the WT. Detailed analysis is given in the main text.

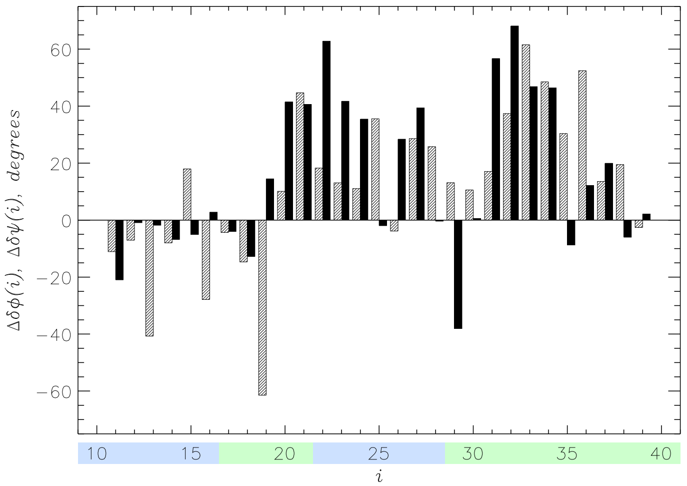

Fig. S11 Differences in the backbone dihedral angle standard deviations, Δδ*φ*(*i*) and Δδ*ψ*(*i*), between MetO and WT Aβ computed for amino acids *i*. We define $\Delta\delta\varphi(i) = \delta\varphi(i) - \delta\varphi(i)_{WT}$ and $\Delta\delta\psi(i) = \delta\psi(i) - \delta\psi(i)_{WT}$, where δ*φ*(*i*) or δ *ψ*(*i*) refer to MetO and $\delta\varphi(i)_{WT}$ or $\delta\psi(i)_{WT}$ - to the WT. Shaded and solid bars represent Δδ*φ*(*i*) and Δδ*ψ*(*i*), respectively. Regions R1-R4 are colored according to Fig. 1a. The figure shows that, except for the N-terminus, fluctuations in MetO Aβ are typically greater than in its WT counterpart.

Free energy of Aβ binding to the DMPC bilayer: To compute the free energy of binding of Aβ peptide to the DMPC bilayer, we used equilibrium replica exchange conformational sampling and MM-GBSA approach. The free energy of a system *G* is

given by Eq. (1). The description of individual terms in G is given in the main text, whereas their values are listed in Table S5.

Table S5 Contributions of individual free energy terms to binding affinity[a,b,c]

	**WT Aβ**	**MetO Aβ**	**WT Aβ+DMPC**	**MetO Aβ+DMPC**
E_{mm}, kcal/mol	361±5	345±6	5669±30	5777±33
$G_{solv,p}$, kcal/mol	-529±4	-536±6	-2567±30	-2688±33
$G_{solv,ap}$, kcal/mol	16±0	17±0	125±1	128±0
TS, kcal/mol	4±0	4±0	4±0	4±0

[a] Free energy contributions are rounded to whole numbers. Terms in $\Delta\Delta G_b$ were computed before rounding.
[b] Free energy contributions cannot be directly used to compute the binding free energies of WT or MetO peptides because of different energy scales in the systems with and without the bilayer. This does not preclude computation of $\Delta\Delta G_b$ because the energy scales for the oxidized and reduced peptides in water or bound to the bilayer were identical.
[c] Uncertainties are standard errors about the mean calculated from n=5 (MetO Aβ) and n=10 (WT Aβ) trajectories.

It is important to check the consistency of computation of solvation free energies $G_{solv,p}$ and $G_{solv,ap}$. The polar solvation free energy $G_{solv,p}$ is calculated using Generalized Born Implicit Solvent model, which offers two sets of parameters for estimating effective Born radius, denoted as OBC I and OBC II [7]. In addition, we tested the effect of changing the nonpolar surface tension coefficient γ from γ_1=0.005 to γ_2=0.02 kcal/mol/AA^2 [8]. We found that depending on the specific combination of parameters, the difference in the free energy of binding between MetO and WT peptides $\Delta\Delta G_b$ (Eq. (3)) is 17 kcal/mol (OBC II and γ_1, used in Table S5), 22 kcal/mol (OBC II and γ_2), 17 kcal/mol (OBC I and γ_1), and 23 kcal/mol (OBC I and γ_2). These computations indicate that irrespective of specific choice of solvation parameters, oxidation decreases the affinity of Aβ binding to the DMPC bilayer.

Our computations of $\Delta\Delta G_b$ revealed a vanishing entropic contribution $-T\Delta\Delta S\approx 0$ kcal/mol. This result suggests that oxidation induces similar changes in entropy in the MetO peptide bound to the bilayer and solvated in lipid-free water.

References

[1] Wang, L., Friesner, R. A., and Berne, B. J. (2011) Replica Exchange with Solute Scaling: A More Efficient Version of Replica Exchange with Solute Tempering (REST2). *J. Phys. Chem B* **115**, 9431–9438.
[2] Smith, A., Lockhart, C., and Klimov, D.K. (2016) Does Replica Exchange with Solute Tempering efficiently sample Aβ peptide conformational ensembles? *J. Chem. Theor. Comput.,* **12**, 5201–5214.
[3] Kim, J., Keyes, T., and Straub, J. E. (2009) Replica exchange statistical temperature Monte Carlo. *J. Chem. Phys.* **130**, 124112.
[4] Han, M. and Hansmann, U. H. E. (2011) Replica exchange molecular dynamics of the thermodynamics of fibril growth of Alzheimer's Aβ42 peptide. *J. Chem. Phys.* **135**, 065101.

[5] Eisenberg, D., Weiss, R. M., Terwilliger, T. C., and Wilcox, W. (1982) Hydrophobic moments and protein structure. *Faraday Symp. Chem. Soc.* **17,** 109-120.
[6] Wimley, W. C., Creamer, T. P., and White, S. H. (1996) Solvation energies of amino acid side chains and backbone in a family of host-guest pentapeptides. *Biochem.* **35**, 5109-5124.
[7] Onufriev, A., Bashford, D., and Case, D. A. (2004) Exploring protein native states and large-scale conformational changes with a modified Generalized Born model. *Proteins: Struct. Func. Gen.* **55**, 383-394.
[8] Knight, J. L. and Brooks III, C. L. (2011) Surveying implicit solvent models for estimating small molecule absolute hydration free energies. *J. Comput Chem.* **32**, 2909–2923.
